# Skeletal Muscle Measurements Based on Abdominal Computerized Tomography (CT) Predict Risk of Osteoporosis in Incident Hemodialysis Patients

**DOI:** 10.3390/jcm13247696

**Published:** 2024-12-17

**Authors:** Hwajin Park, Suyeon Han, Yunkyeong Hwang, Wonjung Choi, Yu Ah Hong, Yoon-Kyung Chang

**Affiliations:** 1 Division of Nephrology, Department of Internal Medicine, Daejeon St. Mary’s Hospital, Daejeon 34943, Republic of Korea; hwajin_2020@catholic.ac.kr (H.P.); sooyaa1208@gmail.com (S.H.); hyk5210@gmail.com (Y.H.); 1jungchoi@gmail.com (W.C.); amorfati@catholic.ac.kr (Y.A.H.); 2 The College of Medicine, The Catholic University of Korea, Seoul 06591, Republic of Korea

**Keywords:** osteoporosis, hemodialysis, sarcopenia, skeletal muscle area, cross-sectional CT image

## Abstract

**Objective**: Osteoporosis is prevalent in patients with chronic kidney disease (CKD), with risk increasing as CKD progresses, subsequently elevating fracture risk. While previous studies have shown a link between low skeletal muscle mass and osteoporosis in the general population, there is limited research exploring this relationship in patients with advanced CKD (stages 3-5D). This study aimed to evaluate whether skeletal muscle area (SMA), as measured by abdominal CT, is correlated with bone mineral density (BMD) in advanced CKD patients beginning hemodialysis. **Methods**: This single-center, retrospective cohort study included patients who started maintenance hemodialysis at Daejeon St. Mary’s Hospital from January 2018 to September 2021. Patients who underwent abdominal CT and BMD assessments within three months of dialysis initiation were enrolled, resulting in a sample of 87 individuals. Baseline characteristics were analyzed, with patients stratified by sex and SMA quartiles. Pearson’s correlation and multivariate regression analyses were conducted to the relationship between SMA and BMD T-scores. **Results**: The study cohort had an average age of 65.4 years, with 52.9% of participants being male. Male patients exhibited significantly higher SMA and BMD T-scores in both the lumbar spine and femur compared to female patients. SMA showed the strongest positive correlation with BMD at both sites (lumbar spine, r = 0.424; femur, r = 0.514; *p* < 0.001). Multivariate analysis identified SMA as an independent positive predictor of BMD, while alkaline phosphatase (ALP) was independently associated with lower femur BMD. In the SMA-based subgroup analysis, patients with lower SMA had significantly lower BMD T-scores and a higher risk of osteoporosis. Logistic regression indicated that patients in the lowest SMA quartile had substantially increased odds of osteoporosis compared to those in the highest quartile, with an adjusted odds ratio of 30.59 (*p* = 0.008). **Conclusions**: Lower skeletal muscle mass is significantly associated with lower bone density and a higher risk of osteoporosis in advanced CKD patients initiating hemodialysis. SMA, as measured by abdominal CT, may serve as a useful marker for identifying patients at elevated osteoporosis risk in this population.

## 1. Introduction

Sarcopenia, the loss of muscle mass, is known to be associated with decreased muscle strength, low physical activity, and higher mortality rates, usually along with aging. In advanced CKD (stages 3-5D) patients, sarcopenia is highly prevalent, independently of other conditions [1]. Sarcopenia in advanced CKD patients results from the increased breakdown of protein and decreased protein synthesis because of dietary restriction, systemic inflammation, hormonal derangements, comorbidities, dialysis, and other consequences of uremic toxicity [2,3]. Advanced CKD patients with sarcopenia are more predisposed to experience low physical performance, disability, and frailty, and these conditions eventually lead to increased mortality [3,4,5].

Osteoporosis and fractures are common in advanced CKD patients. In view of frailty, disability, and malnutrition, sarcopenia and osteoporosis are associated with major mortality risks in advanced CKD patients and the general aging population. Many studies have suggested that skeletal muscle has a positive relationship with bone through several muscle–bone mechanisms, and both share common risk factors such as aging, sex, and hormonal deficiencies [5,6,7,8]. Several observational studies have shown a strong relationship between low skeletal muscle mass and osteoporosis in the general population [9,10,11]. However, such studies have rarely been conducted for advanced CKD or ESKD patients, despite their higher prevalence of sarcopenia and osteoporosis, and poorer survival prognosis than the general population [12].

Furthermore, as an objective assessment of skeletal muscle mass, an abdominal computerized tomography (CT) scan is regarded as one of the gold-standard tools for body composition measurement, particularly skeletal muscle mass, by transverse-section analysis. Previous studies have indicated that the cross-sectional skeletal muscle area at the level of the third lumbar vertebra (L3) well represents the total body skeletal muscle mass and can be used for sarcopenia assessment in the general population [13,14]. In addition, Cheng-bin et al. demonstrated that the L3-based skeletal muscle index (SMI) and psoas muscle index (PMI) could be used to predict osteoporosis in the general population, showing that they were closely associated with the FRAX score for major osteoporotic fractures [15].

Thus, the authors aimed to determine whether skeletal muscle mass measured by abdominal CT scan at the third lumbar spine area correlates clinically with bone mineral density (BMD), and whether the skeletal muscle mass of the L3 area predicts the risk of osteoporosis occurrence in advanced CKD patients initiating hemodialysis, by retrospective cohort analysis.

## 2. Materials and Methods

### 2.1. Study Population and Study Design

Patients who started hemodialysis (HD) for maintenance dialysis at Daejeon St. Mary Hospital between January 2018 and September 2021 were investigated retrospectively. Among them, those who had undergone abdominal (CT) and bone mineral density (BMD) measurements within 3 months at the initiation of dialysis treatment were enrolled. Patients diagnosed with acute kidney injury, those undergoing hemodialysis for <3 months, aged < 18 years, with <3 months of survival, or with malignancy were excluded. A total of 87 patients were included in this study. All patients followed the standard hemodialysis schedule of three sessions per week, with a weekend break.

This single-center retrospective cohort study was conducted in accordance with the principles of the Declaration of Helsinki and received approval from the Institutional Review Board of the College of Medicine, the Catholic University of Korea (IRB no. DC23RASI0058).

For baseline characteristics analysis, all patients were compared by sex.

For regression analysis between skeletal muscle mass and T-score on BMD, all patients were categorized into four groups based on the interquartile range of skeletal muscle area (SMA, cm^2^) values obtained in abdominal CT scan (25th percentile of the SMA value: 100.31(cm^2^); 50th percentile of the SMA value: 122.70 (cm^2^); 75th percentile of the SMA value: 143.24 (cm^2^)).

### 2.2. Data Collections and Measurements

Patient information was collected from the medical records documented on the closest date within one month before the first HD session: age, sex, initial HD date, body mass index (BMI), body weight and height, medication, and history of the cause of ESKD. Blood and biochemical data were quoted from samples collected just before the initiation of the first HD session. The abdominal CT scan and BMD were conducted from the day after initiating hemodialysis to 3 months after the first HD session, while the patients were stabilized from acute complications of end-stage kidney disease (ESKD).

**Figure 1:**
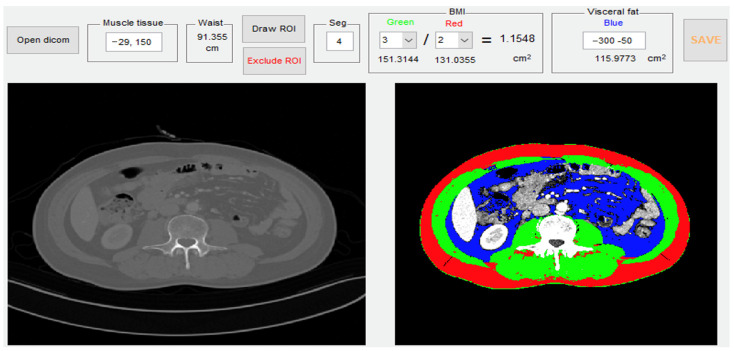
The measurements of body composition using abdominal CT scan image. SMA (green, cm^2^), SFA (red, cm^2^), and VFA (blue, cm^2^). MATLAB version R2014a (Mathworks Inc., Natick, MA, USA) (https://sourceforge.net/projects/muscle-fat-area-measurement (accessed on 1 September 2023)).

For body composition measurement, abdominal CT scan images were used. The analysis was conducted using open-source software to identify skeletal muscle area (SMA), subcutaneous fat area (SFA), and visceral fat area (VFA) in CT images (Figure 1). MATLAB version R2014a (Mathworks Inc, Natick, MA) was used (https://sourceforge.net/projects/muscle-fat-area-measurement (accessed on 1 September 2023)) [16]. The abdominal CT transverse view image at the upper border of lumbar level 3 (LS3) single cross-section was obtained, and semi-automatic software was utilized with cut-off levels (−300 to −50 HU for fat tissue; −29 to +150 HU for muscle) to extract the SMA, SFA, and VFA. The abdominal CT examinations were conducted using multidetector row CT (MDCT) scanners, specifically Simens SOMATOM Definition Flash (dual source 128) and Simens SOMATOM Force (dual source 192). The scanning parameters were set at 90 kVp, 109–120 mAs, and a 3 mm slice thickness.

BMD was assessed at the lumbar spine (L1–L4) and the proximal femur, including the total and neck regions, using dual-energy X-ray absorptiometry (model: Horizon W (S/N304140M), HOLOGIC). To improve the accuracy of the interpretation, the T-score at the site of fracture or vertebroplasty was excluded from analysis.

### 2.3. Definitions and Indexes

Using abdominal CT scan images, body compositions were represented as follows: skeletal muscle area (SMA, cm^2^) as skeletal muscle mass, subcutaneous fat area (SFA, cm^2^) as subcutaneous fat mass, and visceral fat area (VFA, cm^2^) as visceral fat mass, respectively. Total fat area (TFA, cm^2^) was the sum of SFA and VFA.

Personalized indexes for SMA, VFA, SFA, and TFA were normalized by height squared to calculate the skeletal muscle index (SMI, cm^2^/m^2^), visceral fat index (VFI, cm^2^/m^2^), subcutaneous fat index (SFI, cm^2^/m^2^), and total fat index (TFI, cm^2^/m^2^) [17].

The prognostic nutritional index (PNI) score, a nutritional marker, was calculated as serum albumin (g/L) + 5 × lymphocyte count (10^9^/L) to assess the nutritional status of study patients [18].

Osteoporosis was defined as a T-score less than −2.5 of BMD in the lumbar spine or proximal femur. The traditional osteoporosis risk factors were as follows: hypocalcemia, defined as albumin-corrected serum Ca < 8.5 mg/dL; hyperphosphatemia, defined as serum P > 4.5 mg/dL; vitamin D deficiency, defined as 25(OH)D < 15 ng/mL; and hyperparathyroidism, defined as serum PTH > 65 pg/mL [19].

### 2.4. Statistical Analysis

Continuous variables were presented as mean ± standard deviation and compared using the *t*-test. Categorical variables were expressed as numbers with percentages and compared using the chi-squared test. A *p*-value of <0.05 was considered statistically significant. Pearson’s correlation coefficient and univariate regression analysis were used to evaluate the clinical parameters associated with the lumbar spine and proximal femur BMD. Multivariate regression analysis was subsequently performed, including baseline variables with a *p*-value < 0.05 from the univariate analysis, to identify independent factors influencing each BMD. Comparisons among the quartile SMA groups were evaluated using one-way ANOVA for the continuous measures and the chi-squared test for the categorical measures. Odds ratios (ORs) and 95% confidence intervals (CIs) were calculated by logistic regression analysis to assess the risk of low SMA on the occurrence of osteoporosis, with a *p*-value of <0.05 considered statistically significant. All statistical analyses were carried out using SPSS Statistics version 20.0 (IBM Corporation, Armonk, NY, USA).

## 3. Results

### 3.1. Baseline Characteristics

The baseline characteristics of all 87 patients are shown in Table 1. The mean age of all patients was 65.37 ± 13.76 years, and 46 (52.9%) of 87 patients were male. The mean BMI was 24.15 ± 4.17 kg/m^2^. The most common cause of ESKD was diabetic nephropathy. Most of the patients used a central venous catheter as vascular access at the initiation of hemodialysis. The average ultrafiltration (UF) values during the first and second HD sessions were 0.97 L and 1.19 L, respectively. For body composition analysis, the mean SMA, SMI, SFA, and VFA in all 87 patients were 122.92 ± 29.06 cm^2^, 47.01 ± 8.92 cm^2^/m^2^, 128.08 ± 58.47, and 143.53 ± 93.96 cm^2^, respectively. The mean T-scores of lumbar spine and femur neck BMD were −1.29 ± 1.50 and −1.80 ± 2.13. The Kt/V and URR values for the 23 patients were 1.47 ± 0.31 and 69.78 ± 7.02, respectively. For the 14 male patients, the Kt/V and URR values were 1.31 ± 0.18 and 66.21 ± 4.89, respectively, while for the 9 female patients, the Kt/V and URR values were 1.71 ± 0.32 and 75.33 ± 6.31, respectively.

Patients were compared by sex in Table 1. Male patients showed significantly higher mean body weight (68.05 ± 13.38 vs. 57.23 ± 12.24, *p* < 0.001) and height (166.94 ± 5.31 vs. 154.70 ± 7.74, *p* < 0.001). Female patients showed significantly higher total cholesterol and LDL-cholesterol. Details about the history of medication use are illustrated in Table S1.

For body composition analysis, SMA (137.22 ± 26.48 vs. 106.88 ± 22.98, *p* < 0.001) and SMI (49.06 ± 8.10 vs. 44.72 ± 9.32, *p* = 0.023) were significantly higher in male patients. SFA was significantly higher in female patients compared to males (143.13 ± 62.35 vs. 114.66 ± 51.83, *p* = 0.023). VFA of male patients was higher (162.01 ± 103.11 vs. 122.78 ± 78.65, *p* = 0.051) but statistically insignificant. The mean T-scores of BMD in male patients were significantly higher than in female patients for both the lumbar spine (−0.79 ± 1.24 vs. −1.86 ± 1.58, *p* = 0.001) and proximal femur (−1.66 ± 1.07 vs. −2.54 ± 1.02, *p* < 0.001).

### 3.2. Correlation of Clinical Parameters with T-Scores of Both BMDs

The clinical parameters affecting the T-scores of both BMDs were evaluated by Pearson’s correlation analysis in Table 2. The factors positively correlated with both lumbar spine and femur neck BMD were male sex, body weight, height, SMA, SMI, TFA, and VFA, all of which were statistically significant. The factors negatively correlated with both lumbar spine and femur neck BMD were age and ALP, both statistically significant.

**Figure 2:**
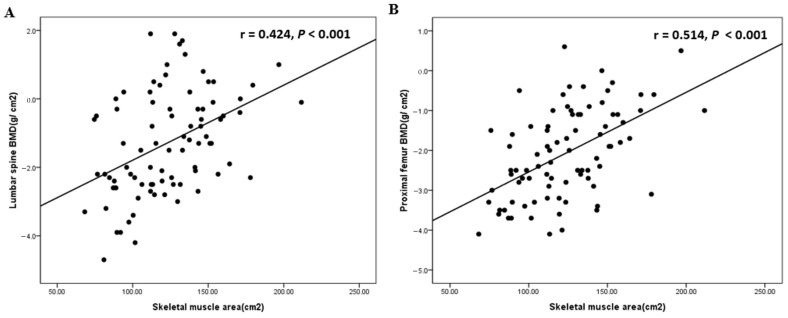
Correlation curves of SMA with lumbar spine BMD (**A**) and femur neck BMD (**B**) (lumbar spine, *r* = 0.424, *p* < 0.001; proximal femur, *r* = 0.514, *p* < 0.001).

Among clinical parameters, SMA showed the highest correlation coefficient with both BMDs (lumbar spine, *r* = 0.424, *p* < 0.001; proximal femur, *r* = 0.514, *p* < 0.001), and the relationship curves between SMA and both BMDs are shown in Figure 2. The relationship curves of SMI, TFA, and VFA with both BMDs are illustrated in Figure S1 and Table S2.

In multivariate linear regression analysis, SMA independently showed a positive correlation with lumbar spine BMD (*p* < 0.001) and proximal femur BMD (*p* = 0.002). Alkaline phosphatase (ALP) showed an independent negative correlation with proximal femur BMD (*p* = 0.003), shown in Table 3. The only variable significantly associated with both BMDs was SMA, and the standardized coefficient β of SMA was the greatest among the significantly affected variables (β = 0.434, β = 0.407).

### 3.3. Subgroup Analysis on Interquartile SMA Groups

All patients were divided into four groups based on the interquartile ranges of measured SMA values: high SMA (1st quartile, more than 143.24 (cm^2^)), moderate SMA (2nd quartile, 122.70~143.24 (cm^2^)), low SMA (3rd quartile, 100.31~122.70 (cm^2^)), and very low SMA (4th quartile, less than 100.31 (cm^2^)) groups.

Comparisons of the clinical characteristics of the four SMA groups are shown in Table 4. There were no significant differences in age, DM (%), albumin, corrected Ca, phosphorus, intact PTH, Vt D deficiency (%), and PNI score among the four groups. There were statistically significant differences in sex (male), body weight, height, BMI, HTN (%), SMI, TFA, SFA, VFA, and T-scores of lumbar spine and proximal femur BMD. Higher SMA groups showed higher values of body composition markers and higher T-scores of lumbar spine and proximal femur BMD, which were statistically significant. The mean T-scores of both BMDs were the lowest in the very low SMA group (lumber spine BMD, −2.26 ± 1.36; proximal femur BMD, −2.81 ± 0.88) in Table 4.

In logistic regression analysis to estimate the impact of measured SMA on the risk of osteoporosis, the odds ratio and confidence interval (95%, CIs) for SMA value (cm^2^) in relation to osteoporosis were 0.96 and 0.94–0.98 (*p* < 0.001). SMA (cm^2^) was associated with a decreased risk for osteoporosis, as shown in Table 5.

Using the high SMA group as the reference, univariable logistic regression analysis for the impact of SMA groups on the risk of osteoporosis showed the following odds ratios (ORs) and confidence intervals (CIs): moderate SMA group OR = 6.33 (95% CI 1.45–27.74, *p* = 0.014), low SMA group OR = 8.44 (95% CI 1.90–37.59, *p* = 0.005), and very low SMA group OR = 40.11 (95% CI 7.17–224.45, *p* < 0.001). In multivariable logistic regression analysis adjusted for age and sex (Model 1), the odds ratios (ORs) and confidence intervals (CIs) were as follows: moderate SMA group OR = 5.24 (95% CI 1.16–23.74, *p* = 0.32), low SMA group OR = 7.17 (95% CI 1.58–32.62 *p* = 0.011), and very low SMA group OR = 34.77 (95% CI 6.12–197.44 *p* < 0.001). In multivariable logistic regression analysis adjusted for age, sex, weight, height, SFA, VFA, comorbidities [DM (%), HTN (%)], corrected Ca (Ca), phosphorous (P), intact PTH, vitamin D deficiency (%), PNI score, total cholesterol, and CRP (Model 2), the odds ratios (ORs) and confidence intervals (CIs) remained statistically significant: moderate SMA group OR = 7.56 (95% CI 1.07–53.46, *p* = 0.028), low SMA group OR = 8.25 (95% CI 1.10–61.91, *p* = 0.040), and very low SMA group OR = 30.59 (95% CI 2.42–387.31, *p* = 0.008), as shown in Table 5.

## 4. Discussion

Chronic kidney disease (CKD) negatively impacts health outcomes such as mortality, quality of life, physical activity, and hospitalization. Patients with CKD are predisposed to systemic musculoskeletal complications, such as CKD-MBD, sarcopenia, and osteoporosis, each of which is a known risk factor for mortality. Even though low skeletal muscle mass or sarcopenia and osteoporosis result mainly from aging-related diseases in the general population, little is known about the interrelationship between sarcopenia and osteoporosis in advanced CKD patients. Patients with advanced stages of CKD (stage 3-5D) often experience idiopathic osteoporosis and renal osteodystrophy. These conditions result in a higher incidence of bone fractures in CKD-5D patients compared to the general population [20]. Furthermore, bone fractures affecting the hip, spine, humerus, and forearm are associated with increased all-cause and CVD mortality in CKD-5D patients [21]. Therefore, it is important to identify high-risk groups for bone fractures and manage them to prevent fractures. According to the 2017 KDIGO CKD-MBD guideline, performing BMD testing is recommended to predict fracture risk in patients with CKD-5D if the results will impact treatment decisions [22].

As mentioned previously, the authors aimed to determine whether SMA measured at the cross-sectional L3 level by CT is clinically correlated with T-scores of BMD in incident hemodialysis patients. The study population had a mean age of 65.37 ± 13.76 years, mean BMI of 24.15 ± 4.17 (kg/m^2^), L-spine T-score of −1.29 ± 1.50 (g/cm^2^), and proximal femur T-score of −1.80 ± 2.13 (g/cm^2^). Compared by sex, female patients showed no differences in age and BMI but had lower body weight and height than males. In body composition parameters, female patients showed significantly lower SMA, SMI, and VFA, but higher SFA. For BMD, female patients showed significantly lower T-scores in the lumbar spine and proximal femur than males (Table 1**)**. Male sex, SMA, SMI, and VFA showed a significant positive correlation with both BMDs in the correlation analysis of clinical parameters with BMD, including epidemiological, laboratory, nutritional, and body composition parameters (Table 2). The correlation coefficient of SMA was higher than that of other parameters (Figure 2). Interestingly, body weight and height showed a correlation with both BMDs, but BMI lost significance. This can be explained by the fact that body weight in incident hemodialysis patients should not be considered lean body weight, as fluid overload may lead to an overestimation of body weight [23]. After adjusting for clinical variables influencing BMD, only SMA was independently associated with both BMDs (Table 3).

The author’s second aim was to determine whether SMA measured at the cross-sectional L3 level by CT could predict the risk of developing osteoporosis in incident hemodialysis patients. In the comparison of the four groups based on the interquartile range of SMA (cm^2^) values, SMA (cm^2^) showed a significant positive association with sex (male), body size (body weight, height, and BMI), body composition parameters (SMA, SMI, TFA, SFA, and VFA), as well as T-scores of lumbar spine and proximal femur BMD. To assess the contributions of measured SMA values to the occurrence of osteoporosis, univariable and multivariable logistic regression analyses were applied to the four groups, with the high SMA group as the reference (Table 5). The measured SMA (cm^2^) itself had an odds ratio (OR) of 0.96 with a 95% confidence interval (CI), indicating a protective effect on the T-score of BMD (*p*-value < 0.0001). Unadjusted analysis showed that the intergroup OR for the occurrence of osteoporosis increased with moderate SMA, OR 6.33; low SMA, OR 8.44; and very low SMA, OR 40.11, all of which were statistically significant. Multivariable logistic regression analysis, adjusted for age and sex (Model 1), with reference values, showed that intergroup OR for the occurrence of osteoporosis increased with moderate SMA, OR 5.24; low SMA, OR 7.17; and very low SMA, OR 34.77, all of which were statistically significant. Multivariable logistic regression analysis, adjusted for all osteoporosis risk factors (age, female sex, weight, height, DM, HTN, CKD-MBD factors, nutritional markers such as PNI score, T-cholesterol, CRP) (Model 2), with reference values, showed intergroup OR for the occurrence of osteoporosis increased with moderate SMA, OR 7.56; low SMA, OR 8.25; and very low SMA, OR 30.59, all of which were statistically significant. These results indicated that measured SMA predicts the risk of occurrence of osteoporosis in incident hemodialysis patients.

A few previous studies have shown a positive correlation between skeletal muscle mass and BMD for non-dialysis-dependent CKD and HD patients. Montenegro et al. measured parameters of muscle mass (LST, lean soft tissue; ASM, appendicular skeletal muscle mass) using DXA in 257 patients with non-diabetic dialysis-CKD in Brazil [24]. They concluded that low skeletal muscle mass was related to low BMD in these patients. In a Japanese study of 50 HD patients, they found that SMI, measured using BIA, had an independent association with BMD [25].

The strength of our study is that we used abdominal CT images for quantifying skeletal muscle mass using semi-automatic software (MATLAB version R2014a) to overcome a limitation of usual measurement tools (BIA, DXA) in incident hemodialysis patients. Non-contrast-enhanced abdominal CT scans have been increasingly used in many studies as a non-invasive and convenient body composition modality [26]. The transverse-sectional skeletal muscle area at the L3 vertebra level strongly correlates with total body skeletal muscle mass and is commonly used as a surrogate marker for skeletal muscle mass [14]. Notably, in CKD/ESKD patients, CT has the advantage of ensuring high accuracy and reproducibility because it is unaffected by hydration status [27]. Additionally, CT can assess the presence of myosteatosis, which is related to muscle dysfunction and poor prognosis, by identifying reduced skeletal muscle mass radiodensity below 33 HU [28,29,30]. Although a limitation of CT is that cut-off points for low muscle mass have not yet been defined for screening sarcopenia, it is still recommended as the gold-standard tool for assessing skeletal muscle mass in CKD/ESKD patients [31]. As a result, our study supports CT as an effective tool for measuring skeletal muscle mass in hemodialysis patients.

There are several hypotheses regarding the relationship between bone health and skeletal muscle mass in ESKD patients. Osteoporosis and sarcopenia share common risk factors such as aging, malnutrition, inflammation, oxidative stress, and vitamin D deficiencies, all of which are highly prevalent in ESKD patients. These conditions often co-exist, and their synergy accelerates the degradation of proteins in muscles and reduces bone mineral density [1,32]. A possible mechanism linking bone and muscle is introduced via myocyte- or osteocyte-secreted molecules. The myokines, such as interleukins (IL-6, IL-7, IL-8, IL-15), leukemia inhibitory factor (LIF), brain-derived neurotrophic factor (BDNF), irisin, musclin, and myostatin, secreted by myocytes, influence bone metabolism. Similarly, the chemokines expressed by osteocytes potentially influence muscle metabolism, regulating muscle growth [7,32,33].

Another discussion point of our study was that the visceral fat area (VFA) was positively associated with both BMDs, as shown in Table 2. Obesity has been traditionally considered to protect the skeleton against osteoporosis and fracture, while visceral fat tissue is known to be metabolically active and closely related to inflammation, oxidative stress, and insulin resistance, which can have deleterious effects on bone. Ching-Ti and colleagues concluded that higher amounts of visceral adipose tissue, measured from abdominal CT imaging, were associated with stronger weight-bearing bone structure, predominantly in women. They explained that the mechanical loading effect of visceral fat tissue on the skeleton increased the overall bone strength rather than the metabolic effect [23,34]. Although there are inconsistent results regarding the effects of VFA on BMD due to the complex relationship between fat and bone, we hope that prospective and large-scale studies will be conducted to clarify the relationship between fat and bone in ESKD patients undergoing hemodialysis.

There are several limitations in our study. First, our sample size was limited and had a short follow-up duration to analyze the prognosis of mortality and fracture incidents. Already, a large portion of patients have osteoporosis. ESKD patients with osteoporosis have a higher risk of all-cause mortality. Further follow-up studies are warranted. Second, even though the study population was confined to incident hemodialysis patients, the prescription for CKD-MBD was not investigated, especially emerging medications for osteoporosis. Third, we were unable to evaluate the Kt/V of stabilized new dialysis patients due to their transfer to other hospitals, and therefore, we could not perform an analysis of the association between Kt/V and osteoporosis. Fourth, skeletal muscle mass, as measured through body composition analysis, reflects only part of sarcopenia. Observations of the results were summarized, but explanations for the relationship between skeletal muscle mass and T-scores of BMD remain insufficient. Further prospective and longitudinal studies on functional scales are needed to promote bone health and predict morbidity and mortality in ESKD patients.

In conclusion, the authors demonstrated that SMA (cm^2^), measured by abdominal CT scan images as a representative of skeletal muscle mass (SMM), correlated best with T-scores of the lumbar spine and proximal femur BMD compared to other clinical parameters. We demonstrated that SMA (cm^2^) independently predicted the risk of osteoporosis in incident hemodialysis patients. Regular assessment of skeletal muscle mass (SMM) might play a major role in preventing mortality among CKD patients. Thus, strategies for preserving skeletal muscle mass, including nutritional supplementation and exercise therapy, should be implemented following the early prediction and diagnosis of sarcopenia and osteoporosis in incident hemodialysis patients.

## Figures and Tables

**Table 1:** Baseline characteristics of all patients and comparisons by sex.

Characteristics	Total (N = 87)	Male (N = 46)	Female (N = 41)	*p*-Value
Age (years)	65.37 ± 13.76	63.8 ± 13.20	67.12 ± 14.33	0.264
Body weight (kg)	62.95 ± 13.89	68.05 ± 13.38	57.23 ± 12.24	<0.001
Height (cm)	161.17 ± 8.97	166.94 ± 5.31	154.70 ± 7.74	<0.001
BMI (kg/m^2^)	24.15 ± 4.17	24.28 ± 3.86	24.60 ± 5.94	0.762
Cause of ESKD, *n* (%)				0.132
Diabetic nephropathy	56 (64.4)	33 (58.9)	23 (56.1)	
Hypertensive nephropathy	9 (10.3)	6 (13.0)	3 (7.3)	
Glomerulonephritis	11 (12.6)	3 (6.5)	8 (19.5)	
Others	11 (12.6)	4 (8.7)	7 (17.1)	
Vascular access at dialysis initiation, *n* (%)				0.166
Central venous catheter	77 (88.5)	38 (82.6)	39 (95.1)	
AVF	8 (9.2)	6 (13.0)	2 (4.9)	
AVG	2 (2.3)	2 (4.3)	0 (0.0)	
Ultrafiltration during first HD session	0.97 ± 0.75	0.99 ± 0.70	0.94 ± 0.81	0.754
Ultrafiltration during second HD session	1.19 ± 0.82	1.14 ± 0.69	1.24 ± 0.95	0.588
Laboratory findings				
Hb (g/dL)	9.35 ± 1.52	9.36 ± 1.65	9.34 ± 1.43	0.954
BUN (mg/dL)	87.38 ± 30.68	91.99 ± 32.54	82.80 ± 27.93	0.138
Cr (mg/dL)	8.26 ± 3.26	8.76 ± 3.37	7.69 ± 3.07	0.128
eGFR (MDRD)	7.21 ± 3.22	7.69 ± 3.59	6.67 ± 2.68	0.140
Albumin (g/dL)	3.57 ± 0.63	3.54 ± 0.64	3.60 ± 0.63	0.665
C-reactive protein (mg/dL)	2.12 ± 5.34	2.09 ± 4.55	2.15 ± 6.15	0.961
Total cholesterol (mg/dL)	149.86 ± 63.48	131.39 ± 40.67	171.84 ± 77.88	0.006
Triglyceride (mg/dL)	137.23 ± 81.48	128.60 ± 73.38	147.31 ± 90.02	0.315
LDL-cholesterol (mg/dL)	82.79 ± 43.24	70.64 ± 30.59	101.05 ± 52.94	0.032
HDL-cholesterol (mg/dL)	42.28 ± 15.60	39.74 ± 17.77	45.31 ± 12.08	0.115
Corrected Ca (mg/dL)	8.35 ± 0.92	8.22 ± 0.84	8.49 ± 0.98	0.177
Phosphate (mg/dL)	5.68 ± 1.85	5.65 ± 1.89	5.71 ± 1.83	0.895
Intact PTH (pg/mL)	212.56 ± 201.21	180.46 ± 107.66	248.78 ± 267.78	0.143
ALP (IU/L)	85.54 ± 42.45	84.57 ± 40.81	86.59 ± 44.62	0.828
Vitamin D total (ng/mL)	10.79 ± 8.02	9.59 ± 7.19	12.23 ± 8.83	0.184
Uric acid (mg/dL)	7.07 ± 2.49	7.27 ± 2.29	6.85 ± 2.72	0.434
PNI score	41.24 ± 7.57	41.05 ± 8.52	41.45 ± 6.45	0.808
Body composition analysis				
Skeletal muscle area (cm^2^)	122.92 ± 29.06	137.22 ± 26.48	106.88 ± 22.98	<0.001
Skeletal muscle index (cm^2^/m^2^)	47.01 ± 8.92	49.06 ± 8.10	44.72 ± 9.32	0.023
Total fat area (cm^2^)	270.49 ± 133.36	276.74 ± 136.98	263.47 ± 130.53	0.646
Subcutaneous fat area (cm^2^)	128.08 ± 58.47	114.66 ± 51.83	143.13 ± 62.35	0.023
Visceral fat area (cm^2^)	143.53 ± 93.96	162.01 ± 103.11	122.78 ± 78.65	0.051
BMD (T-score)				
Lumbar spine BMD (g/cm^2^)	−1.29 ± 1.50	−0.79 ± 1.24	−1.86 ± 1.58	0.001
Proximal femur BMD (g/cm^2^)	−1.80 ± 2.13	−1.66 ± 1.07	−2.54 ± 1.02	<0.001

**Table 2:** Correlation analysis of clinical parameters with T-scores of lumbar BMD and femur BMD.

	Lumbar Spine BMD		Proximal Femur BMD	
	r	*p*-Value	r	*p*-Value
Sex Male, *n* (%)	0.359	0.001	0.391	<0.001
Age (years)	−0.039	0.721	−0.281	0.009
Body weight (kg)	0.441	<0.001	0.465	<0.001
Height (cm)	0.454	<0.001	0.524	<0.001
BMI (kg/m^2^)	0.179	0.096	0.259	0.018
Past medical history, *n* (%)				
Diabetes	0.245	0.022	−0.071	0.521
Hypertension	0.227	0.034	0.166	0.128
Laboratory findings				
Hb (g/dL)	0.172	0.112	0.114	0.300
BUN (mg/dL)	0.084	0.439	0.121	0.268
Cr (mg/dL)	0.097	0.370	0.167	0.128
eGFR (MDRD)	0.009	0.935	−0.051	0.645
Albumin (g/dL)	0.066	0.546	0.028	0.802
C-reactive protein (mg/dL)	−0.029	0.159	−0.187	0.088
Total cholesterol (mg/dL)	−0.044	0.700	0.013	0.909
Triglyceride (mg/dL)	0.134	0.243	0.065	0.575
LDL-cholesterol (mg/dL)	−0.035	0.835	0.106	0.538
HDL-cholesterol (mg/dL)	−0.228	0.044	−0.057	0.623
Corrected Ca (mg/dL)	0.098	0.366	−0.143	0.193
Phosphate (mg/dL)	0.093	0.391	0.202	0.063
Intact PTH (pg/mL)	−0.057	0.611	−0.036	0.753
ALP (IU/L)	−0.016	0.882	−0.242	0.027
Vitamin D total (ng/mL)	−0.206	0.097	−0.077	0.541
Uric acid (mg/dL)	−0.030	0.782	0.034	0.761
PNI score	0.065	0.547	0.056	0.611
Body composition analysis				
Skeletal muscle area (cm^2^)	0.424	<0.001	0.514	<0.001
Skeletal muscle index (cm^2^/m^2^)	0.267	0.012	0.321	0.003
Total fat area (cm^2^)	0.375	<0.001	0.213	0.050
Subcutaneous fat area (cm^2^)	0.204	0.058	0.096	0.382
Visceral fat area (cm^2^)	0.384	<0.001	0.229	0.035

**Table 3:** Multivariate linear regression analysis of clinical parameters for lumbar spine BMD and proximal femur BMD.

	Variables	t	B	β	*p*-Value
Lumbar spine	SMA (cm^2^)	3.796	0.022	0.420	<0.001
Proximal femur	SMA (cm^2^)	3.229	0.015	0.407	0.002
	ALP (IU/L)	−3.060	−0.007	−0.280	0.003

Adjusted for age, sex, height, comorbidities [DM (%), HTN (%)], cause of ESKD (%), corrected Ca, phosphate, ALP, total vitamin D, iPTH, SMA, SFA, VFA.

**Table 4:** Comparison of clinical characteristics of four groups based on interquartile ranges of SMA values.

	High SMA (N = 22)	Moderate SMA (N = 22)	Low SMA (N = 21)	Very Low SMA (N = 22)	
SMA (cm^2^)	1st quartile >143.24	2nd quartile 122.70~143.24	3rd quartile 100.31~122.70	4th quartile <100.31	*p*-value
Age (years)	59.91 ± 10.98	67.14 ± 16.90	66.76 ± 12.38	67.73 ± 13.47	0.197
Sex Male, *n* (%)	18 (81.8)	17 (77.3)	8 (17.4)	3 (13.6)	<0.001
Bwt (kg)	76.88 ± 14.96	66.21 ± 7.90	57.79 ± 7.23	50.70 ± 7.46	<0.001
Ht (cm)	166.78 ± 8.64	165.06 ± 6.36	159.34 ± 7.74	153.43 ± 6.48	<0.001
BMI (kg/m^2^)	27.58 ± 4.22	24.40 ± 3.12	22.78 ± 2.81	22.88 ± 6.97	0.003
Cause of ESKD, *n* (%)					0.451
Diabetic nephropathy	16 (72.7)	17 (77.3)	13 (61.9)	10 (45.5)	
Hypertensive nephropathy	2 (9.1)	3 (13.6)	1 (4.8)	3 (13.6)	
Glomerulonephritis	2 (9.1)	1 (4.5)	3 (14.3)	5 (22.7)	
Others	2 (9.1)	1 (4.5)	4 (19.0)	4 (18.2)	
Laboratory findings					
Corrected Ca (mg/dL)	8.17 ± 0.97	8.14 ± 1.05	8.54 ± 0.70	8.55 ± 0.87	0.273
Phosphate (mg/dL)	5.72 ± 1.73	5.63 ± 1.82	5.74 ± 1.98	5.64 ± 1.98	0.996
Intact PTH (pg/mL)	188.45 ± 109.51	184.60 ± 120.28	196.22 ± 131.27	289.65 ± 356.18	0.305
Vitamin D total (ng/mL)	8.73 ± 7.65	9.62 ± 5.67	13.59 ± 10.37	11.13 ± 7.14	0.314
VitD deficiency (*n* (%))	18 (94.7)	11 (68.8)	14 (66.7)	14 (73.7)	0.126
Hb (g/dL)	9.60 ± 1.40	9.41 ± 1.68	9.02 ± 1.57	9.36 ± 1.56	0.949
BUN (mg/dL)	86.64 ± 33.44	90.71 ± 27.06	95.03 ± 27.24	77.49 ± 33.51	0.781
Cr (mg/dL)	8.46 ± 2.46	8.35 ± 3.91	9.01 ± 3.55	7.24 ± 2.90	0.068
Albumin (g/dL)	3.54 ± 0.70	3.61 ± 0.59	3.69 ± 0.56	3.45 ± 0.68	0.718
C-reactive protein (mg/dL)	0.62 ± 1.27	1.99 ± 4.33	1.50 ± 2.73	4.33 ± 9.01	0.002
Total cholesterol (mg/dL)	154.29 ± 93.52	133.90 ± 38.80	156.80 ± 43.82	154.25 ± 63.04	0.112
Triglyceride (mg/dL)	170.60 ± 100.66	135.85 ± 76.77	116.10 ± 59.66	125.17 ± 78.67	0.419
LDL-cholesterol (mg/dL)	80.10 ± 44.98	72.95 ± 33.20	101.02 ± 46.49	79.62 ± 62.05	0.271
HDL-cholesterol (mg/dL)	35.21 ± 9.68	40.45 ± 22. 12	45.41 ± 11.25	48.37 ± 14.00	0.337
ALP(IU/L)	94.38 ± 46.28	81.76 ± 39.77	85.29 ± 44.34	80.95 ± 40.91	0.802
Uric acid (mg/dL)	7.23 ± 1.67	7.59 ± 2.66	6.76 ± 3.17	6.68 ± 2.36	0.199
PNI score	42.66 ± 7.90	40.01 ± 8.20	43.31 ± 6.51	39.06 ± 7.57	0.192
Body composition analysis					
SMA (cm^2^)	160.28 ± 17.99	131.40 ± 6.48	112.37 ± 6.00	87.15 ± 8.38	<0.001
SMI (cm^2^/m^2^)	57.76 ± 5.90	48.40 ± 3.76	44.55 ± 4.95	37.24 ± 4.92	<0.001
TFA (cm^2^)	354.20 ± 156.58	271.47 ± 113.12	245.51 ± 107.27	209.63 ± 112.79	0.002
SFA (cm^2^)	156.88 ± 70.41	123.01 ± 54.05	128.02 ± 46.05	104.39 ± 51.17	0.025
VFA (cm^2^)	197.19 ± 108.87	148.45 ± 93.32	117.48 ± 80.53	109.79 ± 67.12	0.007
BMD T-score (g/cm^2^)					
Lumbar spine	−0.65 ± 1.03	−0.77 ± 1.51	−1.51 ± 1.56	−2.26 ± 1.36	0.001
Proximal femur	−1.39 ± 1.04	−1.69 ± 1.02	−2.46 ± 1.00	−2.81 ± 0.88	<0.001

**Table 5:** Univariable and multivariable logistic regression analysis of measured SMA values for osteoporosis occurrence.

Variables	Reference	OR	95% CI	*p*-Value
SMA (cm^2^)		0.96	0.94–0.98	<0.001
Group				
Unadjusted	High SMA	1		
	Moderate SMA	6.33	1.45–27.74	0.014
	Low SMA	8.44	1.90–37.59	0.005
	Very low SMA	40.11	7.17–224.45	<0.001
* Model 1	High SMA	1		
	Moderate SMA	5.24	1.16–23.74	0.032
	Low SMA	7.17	1.58–32.62	0.011
	Very low SMA	34.77	6.12–197.44	<0.001
** Model 2	High SMA	1		
	Moderate SMA	7.56	1.07–53.46	0.043
	Low SMA	8.25	1.10–61.91	0.040
	Very low SMA	30.59	2.42–387.31	0.008

* Model 1: Adjusted for age and sex. ** Model 2: Adjusted for age, sex, height, subcutaneous fat area (SFA), visceral fat area (VFA), comorbidities [DM (%), HTN (%)], cause of ESKD (%), PNI score, corrected Ca, P, intact PTH, vitamin D deficiency (%), total cholesterol, CRP.

## Data Availability

All data are reported in the article.

## Supplementary Materials

The following supporting information can be downloaded at https://www.mdpi.com/article/10.3390/jcm13247696/s1, Figure S1: Correlation between clinical markers and BMD. Table S1. Correlation analysis between each BMD and the measured values from the body composition analysis. Table S2. Correlation analysis between each BMD and the measured values from the body composition analysis.
