# Supplementary material for: Skeletal Muscle Measurements Based on Abdominal Computerized Tomography (CT) Predict Risk of Osteoporosis in Incident Hemodialysis Patients
Source: J Clin Med. 2024 Dec 17;13(24):7696. doi: 10.3390/jcm13247696 (PMC11680026; doi:10.3390/jcm13247696)
Supplement: Supplementary file 1 [file jcm-13-07696-s001.zip › jcm-3364584-supplementary.pdf]

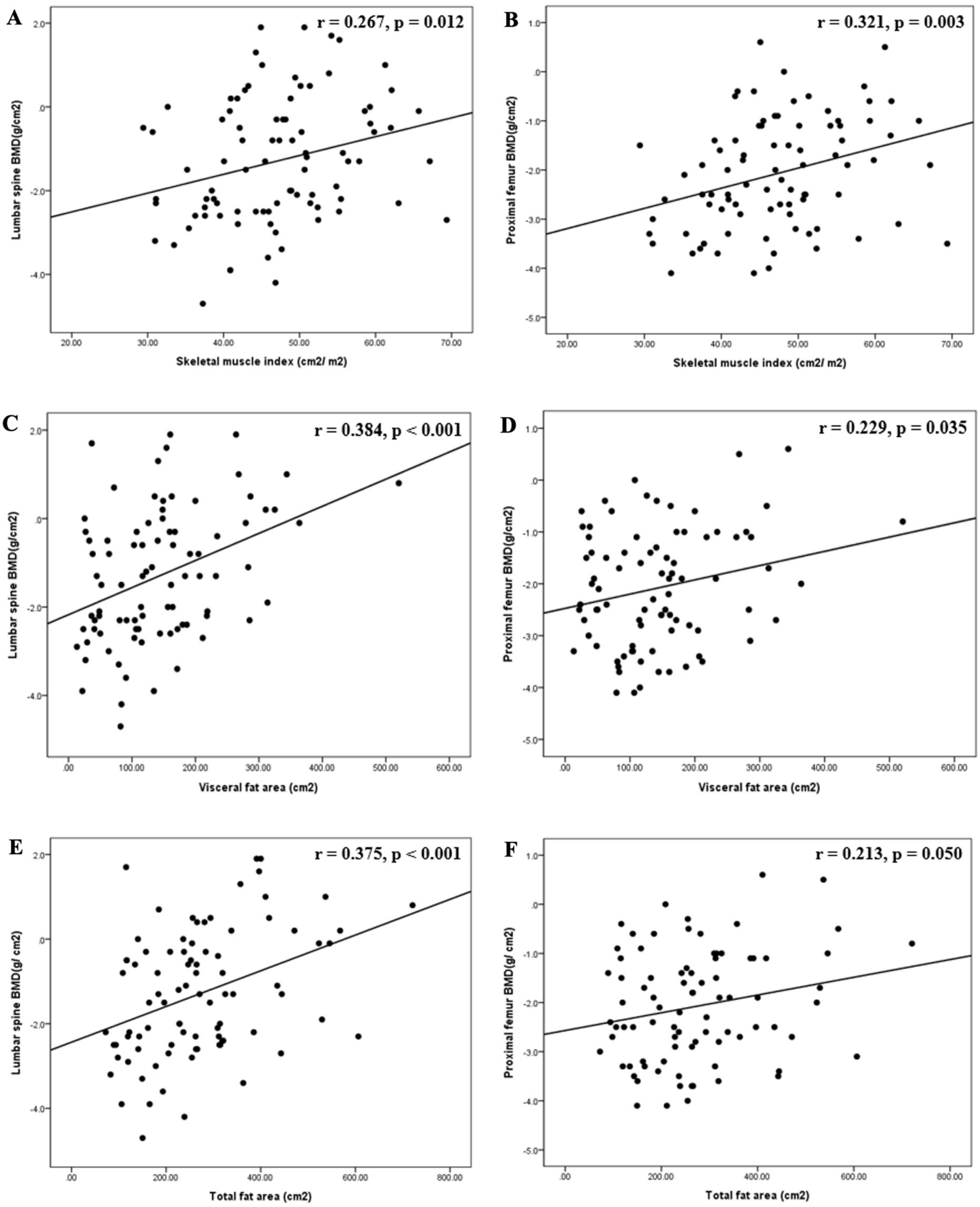

Figure S1. (A) Correlation between SMI and lumbar spine BMD. (B) Correlation between SMI and proximal femur BMD. (C) Correlation between TFA and lumbar spine BMD. (D) Correlation between TFA and proximal femur BMD. (E) Correlation between VFA and lumbar spine BMD. (F) Correlation between VFA and proximal femur BMD.

Table S1. The medication history of study participants and a comparison of medication use among the four SMA groups.

	Total (N=87)	High SMA (N=21)	Moderate SMA (N=22)	Low SMA (N=21)	Very Low SMA (N=22)	*p*-value
**Medication, n(%)**						
RAS-Blocker	42(48.8)	13(59.1)	7(31.8)	10(47.6)	12(57.1)	0.254
B-Blocker	41(47.7)	14(63.6)	8(36.4)	9(42.9)	10(47.6)	0.312
Calcium Channel Blocker	63(73.3)	19(86.4)	16(72.7)	13(61.9)	15(71.4)	0.341
Diuretics	60(69.8)	16(72.7)	15(68.2)	16(76.2)	13(61.9)	0.767
Oral Hypoglycemic Agent	49(57.0)	15(68.2)	15(68.2)	8(38.1)	11(52.4)	0.139
Insulin	22(25.6)	12(54.5)	5(22.7)	4(19.0)	1(4.8)	0.002
Steroid	8(9.3)	1(4.5)	2(9.1)	2(9.5)	3(14.3)	0.725
**Osteoporosis drug, n(%)**						0.586
Vitamin D Derivative & Ca supplement	24(39.5)	7(31.8)	11(50.0)	10(47.6)	6(28.6)	
Bone Resorption Inhibitor	2(2.3)	1(4.5)	0(0.0)	0(0.0)	1(4.8)	

Table S2. Correlation analysis between each BMD and the measured values from the body composition analysis.

	Lumbar spine		Proximal femur	
	**r**	***p*-value**	**r**	***p*-value**
**Body composition analysis**				
Skeletal muscle area(cm^2)	0.424	< 0.001	0.514	< 0.001
Skeletal muscle index(cm^2/ m^2)	0.267	0.012	0.321	0.003
Total fat area(cm^2)	0.375	< 0.001	0.213	0.050
Total fat index(cm^2/ m^2)	0.256	0.017	0.078	0.480
Subcutaneous fat area(cm^2)	0.204	0.058	0.096	0.382
Subcutaneous fat index(cm^2/ m^2)	0.086	0.429	-0.046	0.678
Visceral fat area(cm^2)	0.384	< 0.001	0.229	0.035
Visceral fat index(cm^2/ m^2)	0.314	0.003	0.144	0.188
